# A Novel Bio-Inspired Optimization Algorithm Design for Wind Power Engineering Applications Time-Series Forecasting

**DOI:** 10.3390/biomimetics8030321

**Published:** 2023-07-20

**Authors:** Faten Khalid Karim, Doaa Sami Khafaga, Marwa M. Eid, S. K. Towfek, Hend K. Alkahtani

**Affiliations:** 1 Department of Computer Sciences, College of Computer and Information Sciences, Princess Nourah Bint Abdulrahman University, P.O. Box 84428, Riyadh 11671, Saudi Arabia; 2 Faculty of Artificial Intelligence, Delta University for Science and Technology, Mansoura 35712, Egypt; 3 Computer Science and Intelligent Systems Research Center, Blacksburg, VA 24060, USA; 4 Department of Communications and Electronics, Delta Higher Institute of Engineering and Technology, Mansoura 35111, Egypt; 5 Department of Information Systems, College of Computer and Information Sciences, Princess Nourah Bint Abdulrahman University, P.O. Box 84428, Riyadh 11671, Saudi Arabia

**Keywords:** forecasting wind power, Al-Biruni Earth Radius, metaheuristic algorithm, artificial intelligence

## Abstract

Wind patterns can change due to climate change, causing more storms, hurricanes, and quiet spells. These changes can dramatically affect wind power system performance and predictability. Researchers and practitioners are creating more advanced wind power forecasting algorithms that combine more parameters and data sources. Advanced numerical weather prediction models, machine learning techniques, and real-time meteorological sensor and satellite data are used. This paper proposes a Recurrent Neural Network (RNN) forecasting model incorporating a Dynamic Fitness Al-Biruni Earth Radius (DFBER) algorithm to predict wind power data patterns. The performance of this model is compared with several other popular models, including BER, Jaya Algorithm (JAYA), Fire Hawk Optimizer (FHO), Whale Optimization Algorithm (WOA), Grey Wolf Optimizer (GWO), and Particle Swarm Optimization (PSO)-based models. The evaluation is done using various metrics such as relative root mean squared error (RRMSE), Nash Sutcliffe Efficiency (NSE), mean absolute error (MAE), mean bias error (MBE), Pearson’s correlation coefficient (r), coefficient of determination (R2), and determination agreement (WI). According to the evaluation metrics and analysis presented in the study, the proposed RNN-DFBER-based model outperforms the other models considered. This suggests that the RNN model, combined with the DFBER algorithm, predicts wind power data patterns more effectively than the alternative models. To support the findings, visualizations are provided to demonstrate the effectiveness of the RNN-DFBER model. Additionally, statistical analyses, such as the ANOVA test and the Wilcoxon Signed-Rank test, are conducted to assess the significance and reliability of the results.

## 1. Introduction

Wind energy generation has grown 17% year since 2021. Power grid stability depends on wind speed. Wind variability complicates predictions. New technologies have improved wind speed forecasting with hybrid models and methods. Statistical and artificial intelligence technologies, particularly artificial neural networks, have improved results. Identifying the fundamental aspects affecting forecasting and providing a basis for estimation with artificial neural network models are concerns. The study in [1] classifies modern forecasting models by input model type, pre-and post-processing, artificial neural network model, prediction horizon, steps ahead number, and assessment metric. The research shows that artificial neural network (ANN)-based models can accurately anticipate wind speeds and pinpoint a power plant’s prospective wind use site.

The electricity grid has been faced with obstacles due to the randomness and instability of wind energy, and the connected amount of wind energy poses difficulty in network distribution. For a long time, artificial intelligence has been utilized to anticipate variables in a wide variety of applications, including water desalination [2], transformer faults [3], T-shape antennas [4], engineering problems [5], direct normal irradiation [6], and most notably wind speed forecasting [7].

Utilizing the Deep Neural Networks’ Temporal Convolutional Network (TCN) algorithm, the purpose of the investigation in [8] was to make a long-term (24–72-h ahead) prediction of wind power with a MAPE that was less than 10%. In our experiment, they carried out TCN model pretraining by using historical data on the weather in addition to the power generation outputs of a wind turbine located at a Scada wind power facility in Turkey. The results of the experiments showed that the MAPE for predicting wind power over 72 h was 5.13%, which is sufficient given the limitations of our research. Ultimately, they compared the efficiency of four different DLN-based prediction models for power forecasting. These models were the recurrent neural network (RNN), the gated recurrence unit (GRU), the TCN, and the long short-term memory (LSTM). According to the results of our validation, the TCN is superior to the other three models for predicting wind power in terms of the volume of data input, the consistency of error reduction, and the accuracy of the forecast.

Wind energy is promising. Wind power generation forecasting is essential for smart grid supply-demand balancing. Wind power’s significant variability and intermittent nature make forecasting difficult. The work in [9] develops data-driven wind power generation models. Importantly, the following elements list this work’s important contributions. First, they test upgraded machine learning models for univariate wind power time-series forecasting. We used Bayesian optimization (BO) to optimize hyperparameters of Gaussian process regression (GPR), Support Vector Regression (SVR) with different kernels, and ensemble learning (ES) models (Boosted trees and Bagged trees) and examined their forecasting performance. Second, dynamic information has been added to the models to improve prediction. Lagged measures capture time evolution in model design. More input factors like wind speed and direction increase wind prediction performance. Three wind turbines in France, Turkey, and Kaggle are utilized to test model efficiency. Lagged data and input variables improve wind power forecasts. Optimized GPR and ensemble models also outperformed other machine learning models.

Wind energy has many installations worldwide. Wind speed data power generation prediction accuracy seriously challenges power system regulation and safety. Power system dispatching has various time points relating to area conditions. Power grid dispatching relies on precise wind turbine generation capacity predictions. Wind speed is erratic and intermittent, affecting data quality. The research in [10] proposes a neural network approach that processes wind speed data and predicts electricity generation using BP and Newton interpolation mathematical function methods. BP neural networks handle the hidden layer connection weight learning problem in multi-layer feedforward neural networks. This research examines wind speed data at different heights in the same area at a space scale. The proposed method is 3.1% more accurate than the standard support vector machine method.

On the other hand, models that are based on ANN are able to operate with nonlinear models and do not require prior knowledge of a mathematical model in order to function properly. In addition, some hybrid models forecast wind speed with the use of artificial intelligence applied to time series [11,12]. These models are beneficial because they provide essential information on how to utilize the wind potential of a particular place for the probable construction of a wind power plant by gaining an understanding of future wind speed values [13]. In addition, these models have a fault tolerance and an adaptation of inline measurements. As a result, it is recommended that a substantial amount of data be used in the time series to train the network and produce better results in wind forecasting [14].

The rapid growth of wind power installations has led to increased integration of wind power into the grid [15]. However, this integration brings challenges related to power quality, grid stability, dispatching, and overall power system operation. These challenges are further amplified by the uncertainties associated with wind power generation [16,17]. To ensure the reliable functioning of the power grid, it is crucial to regulate the time series distribution of energy. However, wind power’s nonlinear and unpredictable nature makes achieving this goal difficult. To overcome these challenges, researchers are working towards making wind energy supply more predictable and improving the efficiency and reliability of wind power forecasting. By reducing the unpredictability of wind power, the impact on the power system during the integration of wind power can be minimized. Achieving this requires a solid theoretical foundation and technological advancements that can optimize the operation of the power system, grid dispatching and enhance overall security [18,19]. Through these efforts, it is possible to mitigate the challenges posed by wind power integration and ensure a more stable and secure power grid in the future.

Bio-inspired optimization algorithms handle complex optimization issues by mimicking nature. Evolution, swarm intelligence, and natural selection inspire these strategies. Bio-inspired optimization algorithms solve complicated engineering issues by mimicking nature’s adaptive and efficient solutions [20]. Genetic Algorithm (GA) is a popular bio-inspired optimization algorithm. GA iteratively evolves optimal or near-optimal solutions by selecting, crossover, and mutating a population of candidate solutions. GA has successfully optimized engineering designs and scheduled and estimated parameters. The intelligent technique GA was combined with the Chinese FDS team’s nuclear reactor physics Monte Carlo (MC) code SuperMC for optimal reactor core refilling design in [21]. Particle Swarm Optimization (PSO), another bio-inspired optimization technique, is also prominent. Inspired by bird flocking and fish schooling, PSO uses a population of particles traveling through a problem space, altering their placements based on their experience and other particles. Neural network training, power system optimization, and feature selection use PSO. A hybrid method, such as Aquila Optimizer (AO) with PSO, named IAO, was presented in [22]. Another optimal Ethanol-based method concentration was obtained from the artificial neural network (ANN)-PSO [23]. A complete learning particle swarm optimizer (CLPSO) coupled with local search (LS) is presented to maximize performance by leveraging CLPSO’s robust global search and LS’s fast convergence [24]. Other bio-inspired optimization algorithms include Artificial Bee Colony (ABC) methods, Firefly Algorithm (FA), and Grey Wolf Optimizer (GWO). These algorithms solve optimization problems in image processing, robotics, data mining, and renewable energy systems. Bio-inspired optimization solves complex optimization issues well. By leveraging nature’s wisdom, these algorithms provide new and efficient solutions for various technical applications.

Optimizing parameters in LSTM models for time series prediction is crucial for achieving optimal performance. While LSTM has shown impressive results in this domain, finding the best global solution can be challenging due to the complexity of parameter optimization during model training. As a result, researchers have been studying various methods to improve parameter optimization [25]. One such method is the sparrow search algorithm (SSA), a population intelligence optimization technique introduced in 2020 [26]. SSA is part of a group of emerging metaheuristic algorithms that have gained recognition for their effectiveness in machine learning parameter optimization. It models the behavior of a flock of sparrows as they search for food and avoid predators, aiming to find the optimal solution for a given objective function [27]. Applying SSA optimization to set up network parameters has shown promising results, surpassing the performance of other models in terms of accuracy [28]. For example, SSA has been utilized in estimating ultra-short-term wind power, enhancing the input weight and other parameters of the Dynamic Ensemble Learning Model (DELM) [29]. In another study, short-term wind speed prediction was developed using LSTM and BPNN, and SSA was employed to optimize the prediction model for various complexities in the input sequence [30].

This study suggests a Recurrent Neural Network (RNN) forecasting model based on a proposed Dynamic Fitness Al-Biruni Earth Radius (DFBER) algorithm for capturing and predicting data patterns. We compare its performance with popular models such as BER [31], Jaya Algorithm (JAYA) [32], Fire Hawk Optimizer (FHO) [33], Whale Optimization Algorithm (WOA) [34], Grey Wolf Optimizer (GWO) [35], Particle swarm optimization (PSO) [36], Firefly algorithm (FA) [37], and Genetic Algorithm (GA) [35] based models, utilizing a comprehensive range of evaluation metrics. The evaluation includes metrics like relative root mean squared error (RRMSE), Nash Sutcliffe Efficiency (NSE), mean absolute error (MAE), mean bias error (MBE), Pearson’s correlation coefficient (r), coefficient of determination (R2), and determine agreement (WI), along with considerations of dataset size, estimated and observed values, and arithmetic means of bandwidths. The DFBER-based model demonstrates superior performance compared to the other models, as evident from the evaluation metrics and analysis. Visualizations, including box plots, histograms, residual plots, and heat maps, further validate the model’s ability to capture data patterns. Statistical analyses, such as the ANOVA test and the Wilcoxon Signed-Rank test, strengthen the reliability and significance of the findings.

The main contributions of this research are as follows:

- Providing machine learning-based methodologies to Wind Power time-series forecasting.
- Introducing the optimization-based Recurrent Neural Network (RNN) forecasting model incorporating a Dynamic Fitness Al-Biruni Earth Radius (DFBER) algorithm to predict the wind power data patterns.
- Currently developing an optimized RNN-DFBER-based regression model to enhance the accuracy of predictions using the evaluated dataset.
- Conducting a comparison among different algorithms to determine which one yields the most favorable outcomes.
- Employing Wilcoxon rank-sum and ANOVA tests to assess the potential statistical significance of the optimized RNN-DFBER-based model.
- The RNN-DFBER-based regression model’s flexibility allows it to be tested and customized for various datasets, thanks to its adaptability.

## 2. Materials and Methods

### 2.1. Recurrent Neural Network

**Figure 1:**
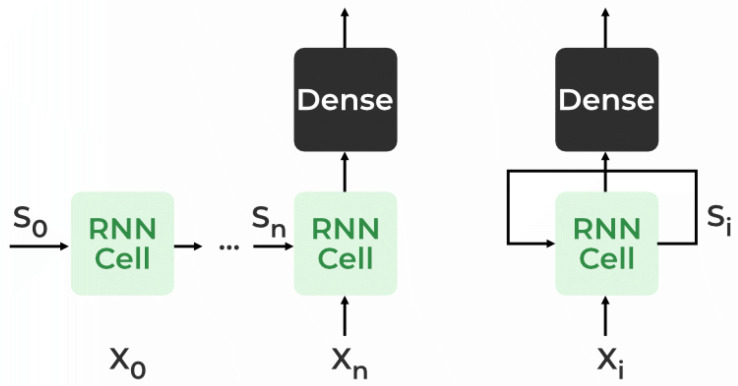
Recurrent neural network architecture.

A Recurrent Neural Network (RNN) is a type of neural network that addresses the need to remember previous information. Unlike traditional neural networks, where inputs and outputs are independent, an RNN utilizes a Hidden Layer to retain and recall previous information. The crucial aspect of an RNN is its Hidden state, which serves as a memory state, storing information about a sequence. By using the same parameters for each input and hidden layer, the RNN simplifies the complexity of parameters compared to other neural networks [38]. Figure 1 shows the RNN architecture. In an RNN, the State matrix (*S*) contains elements ($s_{i}$) representing the state of the network at each time step (*i*). The RNN calculates a hidden state ($H_{i}$) for every input ($X_{i}$) by utilizing the information stored in the State matrix.

### 2.2. Al-Biruni Earth Radius (BER) Algorithm

The first version of the Al-Biruni Earth Radius (BER) optimizes the situation by segmenting the population into groups for exploration and exploitation. Altering the composition of the agent subgroups can help strike a balance between exploitative and exploratory actions. Exploration accounts for 70 percent of the population, denoted as $n_{1}$, while exploitation accounts for 30 percent, denoted as $n_{2}$. The worldwide average levels of the exploration and exploitation groups’ agents’ fitness have improved as a result of the increased number of agents in those groups. The mathematical skills of the exploring crew enable them to locate potentially fruitful places nearby. This can be achieved by persistently looking for more suitable alternatives [31].

Optimization algorithms discover the best agent given constraints. The BER algorithm indicates the population agents as $S=S_{1},S_{2},\ldots ,S_{d}\in R$ (search space) for feature *d*. The objective function, denoted as *F*, is recommended for assessing the agent’s performance. Agents are optimized to find the best agent, denoted as $S^{*}$. The BER algorithm will optimize using the fitness function, as well as the lower and higher limits for each solution, the dimension, and the population size. The original BER algorithm is presented in Algorithm 1.

The solitary explorer in the group will utilize this strategy to look for potentially fruitful new regions to research in the region where they are now situated in order to go closer to the most optimally viable answer. In order to accomplish this objective, one must do research into the numerous options that are available in the surrounding area and choose the alternative that is superior to the others with regard to the effect it has on one’s physical well-being. In order to accomplish this objective, the research that BER has carried out has made use of the equations that are as follows:

(1) $$S\left(t+1\right)=S\left(t\right)+D\left(2r_{2}-1\right)$$

where S(t) is a solution at iteration *t*. With a circle with a diameter of $D=r_{1}\left(S\left(t\right)-1\right)$, the agent will search for promising spots. The *h* parameter is selected within [0,2], and 0<x≤180. The terms *h* and *x* are random variables and follow a uniform distribution. Examples of coefficient vectors include $r_{1}$ and $r_{2}$, and their values can be decided using $r=h\frac{cos(x)}{1-cos(x)}$.

In order to make the most of opportunities, the group responsible for doing so needs to find ways to make the solutions that are already in place even more effective. The BER determines at the end of each round which individuals have achieved the highest levels of fitness and then awards them appropriately. The BER accomplishes its mission of exploitation through the use of two distinct approaches, both of which are detailed in this article. By applying the following equation to help us progress in the right direction, we will be able to move closer to the optimal answer.

(2) $$S\left(t+1\right)=r^{2}\left(S\left(t\right)+D\right),D=r_{3}\left(L\left(t\right)-S\left(t\right)\right)$$

where $r_{3}$ is a random vector calculated based on $r=h\frac{cos(x)}{1-cos(x)}$, which controls the moving to the best solution. L(t) represents the best solution and D is the distance vector.

**Algorithm 1:** AL-Biruni Earth Radius (BER) algorithm1:  **Initialize** BER population and parameters, t=1 2:  **Calculate** objective function $F_{n}$ for each agent $S_{i}$ 3:  **Find** best agent $S^{*}$ 4:  **while**
$t\leq T_{max}$
**do** 5:     **for** ($i=1:i<n_{1}+1$) **do** 6:        **Update** $r_{1}=h_{1}\frac{cos(x)}{1-cos(x)}$, $r_{2}=h_{2}\frac{cos(x)}{1-cos(x)}$ 7:        **Move** to best agent as in Equation (1) 8:     **end for** 9:     **for** ($i=1:i<n_{2}+1$) **do** 10:        **Update** $r=h\frac{cos(x)}{1-cos(x)}$, $r_{3}=h_{3}\frac{cos(x)}{1-cos(x)}$ 11:        **Elitism** of the best agent as in Equation (2) 12:        **Investigating** area around best agent as in Equation (3) 13:        **Select** best agent $S^{*}$ by comparing S(t+1) and $S^{\prime }\left(t+1\right)$ 14:        **if** The best fitness value with no change for two iterations. **then** 15:           **Mutate** solution as in Equation (4) 16:        **end if** 17:     **end for** 18:     **Update** objective function $F_{n}$ for each agent $S_{i}$ 19:     **Find** best agent as $S^{*}$ 20:     **Update** BER parameters, t=t+1 21:  **end while** 22:  **Return** best agent $S^{*}$


Examining the region reveals that the region surrounding the best solution offers the most exciting potential solution. Consequently, several agents hunt for ways to better circumstances by contemplating alternate options that are relatively comparable to the best option. Using the equation that is provided below, the BER completes the procedure that was just described.

(3) $$S^{\prime }\left(t+1\right)=r\left(S^{*}\left(t\right)+k\right),k=1+\frac{2\times t^{2}}{Max_{iter}^{2}}$$

where $S^{*}\left(t\right)$ indicates the best solution. The best solution in this case is determined based on comparing S(t+1) and $S^{\prime }\left(t+1\right)$. In the event that the optimal level of fitness has remained unchanged throughout the course of the previous two iterations, the solution will be modified in line with the equation that is presented below.

(4) $$S\left(t+1\right)=k*z^{2}-h\frac{cos(x)}{1-cos(x)}$$

where *z* has a random value within [0,1] and follow a uniform distribution.

To provide the highest possible level of quality, the BER will select the most suitable alternative for the subsequent cycle. The effectiveness of elitism may hasten the process of multimodal functions converging on one another. By utilizing a mutational strategy and thoroughly assessing all of the members of the exploration group, the BER is able to provide great capabilities for mineral exploration. Exploration allows for a delay in the BER’s convergence. Algorithm 1 has BER pseudo-code. To begin, please provide the size of the BER population, the mutation rate, and the number of iterations. The BER then separates the agents into two distinct groups: the exploratory and the exploitative. The BER method iteratively searches for the best response and automatically modifies the size of each group as it does so. When iterating, the BER will rearrange the responses to ensure that they are diverse and have sufficient depth. In the subsequent cycle, a solution that was developed by the exploration group might be passed on to the exploitation group. During the rigorous and exclusive selection process that is the BER, the leader cannot be replaced.

## 3. Proposed Dynamic Fitness BER Algorithm

This section introduces the innovative Dynamic Fitness Al-Biruni Earth Radius (DFBER) algorithm. This novel algorithm builds upon the original BER algorithm by incorporating it into an adaptable framework. The specifics of the proposed algorithm can be found in Algorithm 2, and further elaboration on its intricacies is provided within this section.

**Algorithm 2:** The proposed DFBER algorithm1:  **Initialize** population and parameters, t=1 2:  **Calculate** fitness function $F_{n}$ for each agent $S_{i}$ 3:  **Find** first, second, and third best agents 4:  **while**
$t\leq T_{max}$
**do** 5:     **if** (For three iterations, the best fitness value is the same) **then** 6:        **Increase** agents in exploration group $\left(n_{1}\right)$ 7:        **Decrease** agents in exploitation group $\left(n_{2}\right)$ 8:     **end if** 9:     **for** ($i=1:i<n_{1}+1$) **do** 10:        **Update**
$r_{1}=h_{1}\frac{cos(x)}{1-cos(x)}$, $r_{2}=h_{2}\frac{cos(x)}{1-cos(x)}$ 11:        **Update** positions of agents as             $S\left(t+1\right)=S\left(t\right)+D\left(2r_{2}-1\right)$ 12:     **end for** 13:     **for** ($i=1:i<n_{2}+1$) **do** 14:        **Update** r = h $\frac{cos(x)}{1-cos(x)}$, $r_{3}=h_{3}\frac{cos(x)}{1-cos(x)}$ 15:        **Update** Fitness $F_{L_{1}}=\frac{F_{L_{1}}}{F_{L_{1}}+F_{L_{2}}+F_{L_{3}}}$ 16:        **Update** Fitness $F_{L_{2}}=\frac{F_{L_{2}}}{F_{L_{1}}+F_{L_{2}}+F_{L_{3}}}$ 17:        **Update** Fitness $F_{L_{3}}=\frac{F_{L_{3}}}{F_{L_{1}}+F_{L_{2}}+F_{L_{3}}}$ 18:        **Calculate**
$D=r_{3}(F_{L_{1}}\times L_{1}\left(t\right)+F_{L_{2}}\times L_{2}\left(t\right)+F_{L_{3}}\times L_{3}\left(t\right)-S\left(t\right)$ 19:        **Elitism** of the best agent as             $S\left(t+1\right)=r^{2}\left(S\left(t\right)+D\right)$ 20:        **Investigating** area around best agent as             $S^{\prime }\left(t+1\right)=r\left(S^{*}\left(t\right)+k\right),k=1+\frac{2\times t^{2}}{Max_{iter}^{2}}$ 21:        **Select** best agent $S^{*}$ by comparing S(t+1) and $S^{\prime }\left(t+1\right)$ 22:        **if** The best fitness value with no change for two iterations. **then** 23:           **Mutate** solution as                $S\left(t+1\right)=k*z^{2}-h\frac{cos(x)}{1-cos(x)}$ 24:        **end if** 25:     **end for** 26:     **Update** fitness function $F_{n}$ for each agent 27:     **Find** best three agents 28:     **Set** first best agent as $S^{*}$ 29:     **Update** parameters, t=t+1 30:  **end while** 31:  **Return** best agent $S^{*}$


### 3.1. Motivation

Optimization problems use populations to represent viable solutions. Each agent in the search space has a parameter vector. Exploitation and exploration groups comprise the prospective solution. An objective function helps the exploitation group enhance the best solution. The exploration group uses the search space to find new places where the best solution may be. In the proposed optimization technique, these two groups share roles and information to quickly find the optimum solution. This partnership avoids local optima and explores the search space efficiently. The suggested optimization technique maintains the balance between exploitation and exploration groups and avoids steady regions in the search space through a dynamic mechanism.

### 3.2. Dynamic Feature of DFBER Algorithm

The proposed DFBER algorithm achieves a balance between exploration and exploitation within the population’s subgroups. It adopts a 70/30 approach, where the population is divided into two groups: exploration, denoted as $n_{1}$, and exploitation, denoted as $n_{2}$. Initially, a larger number of participants in the exploration group facilitates the discovery of new and interesting search regions. As the optimization progresses and individuals in the exploitation group improve their fitness, the overall fitness of the population increases, resulting in a rapid reduction of individuals in the exploration group from 70% to 30%. To ensure convergence, an elitism method is employed, retaining the leader of the process in subsequent populations if a better solution cannot be found. Additionally, if the leader’s fitness remains relatively unchanged for three consecutive iterations, the DFBER algorithm has the flexibility to increase the number of members in the exploration group at any point in time.

The DFBER algorithm balances subgroup exploration and exploitation. Wind power prediction equality and inequality limitations require this balance. Exploration involves searching the solution space broadly to find better solutions, whereas exploitation involves refining and improving the best-known answers in optimization issues. The DFBER algorithm uses exploration and exploitation in population subgroups. This balanced method lets the DFBER algorithm analyze genuine wind power prediction equalities and inequalities. Wind power generating has physical, system, and operational constraints. The DFBER algorithm efficiently navigates the solution space by balancing exploration and exploitation. This method improves wind power estimates by realistically modeling restrictions. The DFBER algorithm’s ability to solve equality and inequality restrictions in wind power projections helps align the optimization process with wind power systems’ real-world limits and requirements.

### 3.3. Fitness Al-Biruni Earth Radius Algorithm

The fitness of the proposed DFBER is formulated as follows. Equation (2) will be updated in the proposed algorithm to include the fitness function property. The fitness functions of $F_{L_{1}}$, $F_{L_{2}}$, and $F_{L_{3}}$ will be calculated for the first best solutions, denoted as $L_{1}\left(t\right)$, $L_{2}\left(t\right)$, and $L_{3}\left(t\right)$, respectively. The final fitness values will be updated as in the following equation to be involved in the proposed algorithm.

(5) $$\mathrm{Fitness}\;\;F_{L_{1}}=\frac{F_{L_{1}}}{F_{L_{1}}+F_{L_{2}}+F_{L_{3}}}$$

(6) $$\mathrm{Fitness}\;\;F_{L_{2}}=\frac{F_{L_{2}}}{F_{L_{1}}+F_{L_{2}}+F_{L_{3}}}$$

(7) $$\mathrm{Fitness}\;\;F_{L_{3}}=\frac{F_{L_{3}}}{F_{L_{1}}+F_{L_{2}}+F_{L_{3}}}$$

Equation (2) of the original BER algorithm will be updated by Equations (8) and (9) in the proposed DFBER algorithm based on the fitness function is as follows.

(8) $$S\left(t+1\right)=r^{2}\left(S\left(t\right)+D\right)$$

For the modified distance vector, D, calculated using $F_{L_{1}}$, $F_{L_{2}}$, and $F_{L_{3}}$ as.

(9) $$D=r_{3}\left(F_{L_{1}}\times L_{1}\left(t\right)+F_{L_{2}}\times L_{2}\left(t\right)+F_{L_{3}}\times L_{3}\left(t\right)-S\left(t\right)\right)$$

where $r_{3}$ is a random vector calculated based on $r=h\frac{cos(x)}{1-cos(x)}$, which controls the moving to the best solution. $L_{1}\left(t\right)$ represents the first best solution, $L_{2}\left(t\right)$ represents the second best solution, and $L_{3}\left(t\right)$ indicates the third best solution. The proposed DFBER algorithm is shown in Algorithm 2 step-by-step.

### 3.4. Complexity Analysis

The DFBER algorithm’s computational complexity, shown in Algorithm 2, can be described using the following expression. We will define the time complexity for a population size of *n*, and a maximum number of iterations denoted as $T_{max}$.

- Initialize population and parameters, t=1:O(1).
- Calculate fitness function for each agent: O(n).
- Finding best three solutions: O(n).
- Updating agents in exploration and exploitation groups: $O(T_{max})$.
- Updating position of current agents in exploration group: $O(T_{max}\times n)$.
- Updating position of current agents in exploitation group by fitness functions: $O(T_{max}\times n)$.
- Updating fitness function for each agent: $O(T_{max})\times n$.
- Finding best fitness: $O(T_{max})$.
- Finding best three solutions: $O(T_{max})$.
- Set first best agent as $S^{*}$: $O(T_{max})$.
- Updating parameters: $O(T_{max})$.
- Updating iterations: $O(T_{max})$.
- Return the best fitness: O(1).

Based on the analysis, the computational complexity of the algorithm can be summarized as follows. The time complexity is estimated to be $O(T_{max}\times n)$ for the case of a population size of *n*. Additionally, the algorithm employs the k-nearest neighbors (kNN) within a fitness function, and the computational complexity of kNN is primarily determined by the number of training samples and the dimensional of the feature space (*d*). Thus, the time complexity becomes $O(T_{max}\times n\times d)$.

### 3.5. Fitness Function

The fitness equation, $F_{n}$, helps the DFBER approach assess a solution’s quality. The expressions for selected features (*v*), the total number of features (*V*), and a classifier’s error rate (Err) employ $F_{n}$ as a variable.

(10) $$F_{n}=h_{1}Err+h_{2}\frac{\left|v\right|}{\left|V\right|}$$

where $h_{2}=1-h_{1}$ indicates the population’s importance of the specified trait, and $h_{1}$ might be any number between [0, 1]. If a selection of features may reduce classification error, the strategy is acceptable. The simple classification method k-nearest neighbor (kNN) is used often. The k-nearest neighbors classifier ensures high-quality attributes in this strategy. The only classifier criterion is the shortest distance between the query instance and the training instances. This experiment uses no K-nearest neighbor models.

## 4. Experimental Results

In the study, the investigation’s findings are thoroughly examined, focusing on the DFBER algorithm. This algorithm is analyzed and compared to other state-of-the-art algorithms including BER [31], Jaya Algorithm (JAYA) [32], Fire Hawk Optimizer (FHO) [33], Whale Optimization Algorithm (WOA) [34], Grey Wolf Optimizer (GWO) [35], Particle swarm optimization (PSO) [36], Firefly algorithm (FA) [37], and Genetic Algorithm (GA) [35]. The DFBER algorithm’s configuration is presented in Table 1. This table details the algorithm’s parameters, which are crucial for understanding its behavior and performance in optimization. The presented parameters include the population size (number of agents), termination criterion (number of iterations), and other important characteristics for selecting significant features from the input dataset.

By comparing the DFBER algorithm with other state-of-the-art algorithms and presenting its configuration, the investigation aims to evaluate its effectiveness and potential advantages. Table 2 presents the setup of the comparative algorithms used in the evaluation. Several factors are considered to ensure a fair evaluation of optimization techniques and parameters. The problem’s search space size, constraints, and objective function are considered. The choice of algorithm parameters can greatly influence its performance. Factors such as convergence speed and the exploration-exploitation trade-off should be considered when tuning parameters for both the problem and the chosen algorithm. The evaluation is conducted over ten runs with different random seeds to ensure fair comparisons. This helps account for the variability in the optimization process and provides a more robust statistical analysis. Furthermore, an appropriate dataset is selected for testing the algorithms. The choice of the dataset should represent the problem domain and provide meaningful insights into the performance of the algorithms. The number of function calls made during optimization is considered to establish a fair computational budget. Each optimizer is run ten times for 80 iterations, with the number of search agents set to 10. Setting a specific computational budget ensures that all compared algorithms have an equal opportunity to explore and exploit the search space within the given limitations. Adopting this approach enables a fair and standardized evaluation, enabling meaningful comparisons between the optimization algorithms and facilitating informed decision-making.

### 4.1. Dataset

The dataset in this context is focused on analyzing energy generation from solar and wind sources in different operating areas. The data used in the analysis is sourced from the National Renewable Energy Laboratory (NREL) and provides information on solar and wind energy generation over specific periods. The dataset includes monthly average wind speeds 50 m above the Earth’s surface regarding wind energy. This wind speed data is collected over 30 years, specifically from January 1984 to December 2013. The wind speed measurements help assess the wind energy potential in different areas [39]. The data is further categorized based on different operating areas:

- Central Operating Area (COA): This refers to a specific region or area where the central energy generation operations occur.
- Eastern Operating Area (EOA): This refers to a specific region or area where energy generation operations are concentrated in the eastern part.
- Southern Operating Area (SOA): This refers to a specific region or area where energy generation operations are concentrated in the southern part.
- Western Operating Area (WOA): This refers to a specific region or area where energy generation operations are concentrated in the western part.

**Figure 2:**
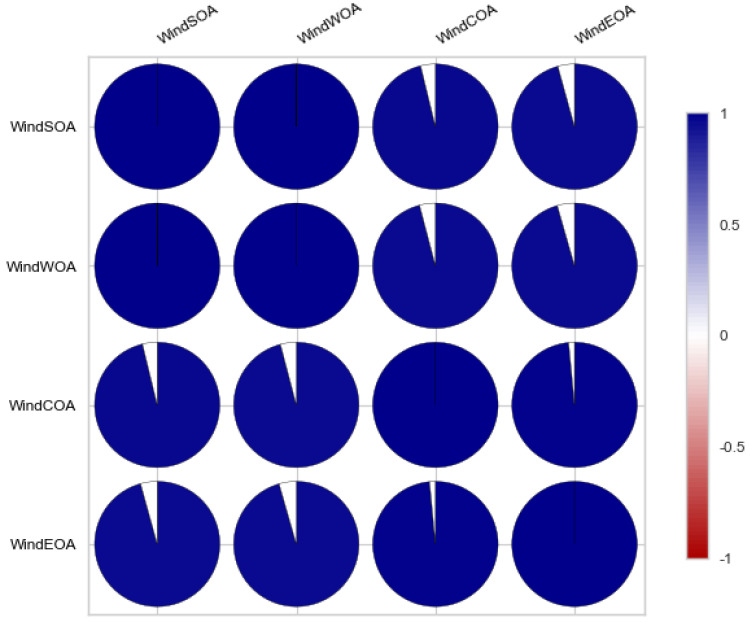
Map of the wind power forecasting dataset.

The dataset is sourced from NREL and associated with the “wind-solar-energy-data identifier”. Figure 2 shows the map of the wind power forecasting dataset. It falls under the theme of Renewable & Alternative Fuels and contains keywords related to wind and solar energy. The dataset is in English and was last modified on 14 October 2020. The publisher of the dataset is the King Abdullah Petroleum Studies and Research Center in Saudi Arabia. The dataset’s availability and classification indicate that it is public data. However, it is noted that the dataset has been discontinued. The dataset specifically focuses on energy generation in Saudi Arabia, as the country’s information indicates. The ISO region associated with the dataset is EMEA (Europe, the Middle East, and Africa).

**Figure 3:**
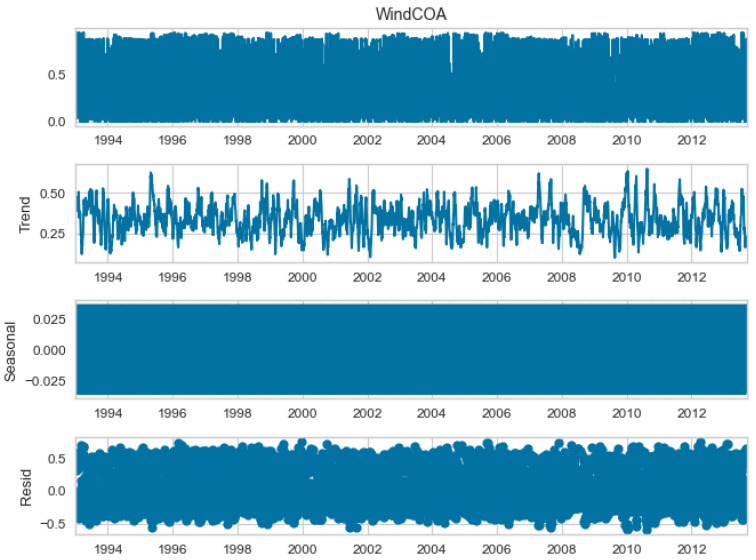
The seasonal decompose for COA of the wind power forecasting dataset.

**Figure 4:**
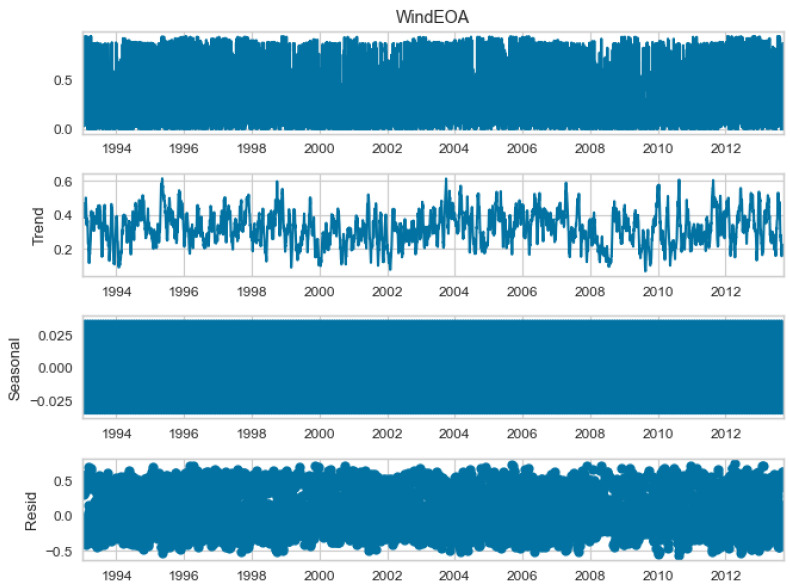
The seasonal decompose for EOA of the wind power forecasting dataset.

**Figure 5:**
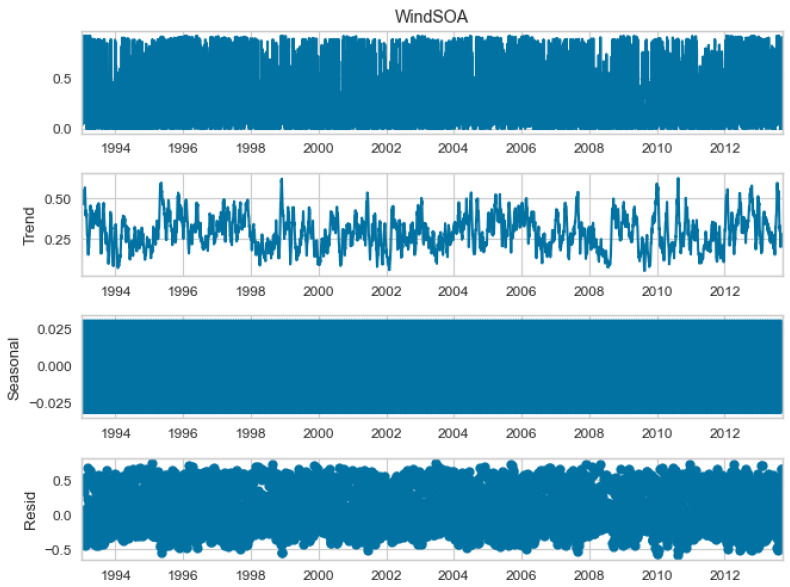
The seasonal decompose for SOA of the wind power forecasting dataset.

**Figure 6:**
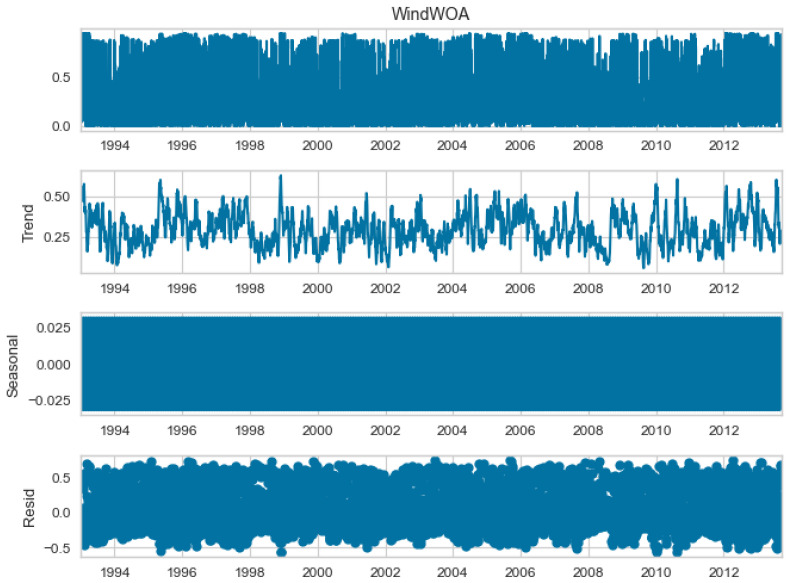
The seasonal decompose for WOA of the wind power forecasting dataset.

The seasonal decomposition of the wind power forecasting dataset provides insights into the individual components of the dataset, namely COA, EOA, SOA, and WOA. Each of these components represents a specific aspect or pattern within the dataset. Figure 3, Figure 4, Figure 5 and Figure 6 offer a visual representation of the seasonal decomposition for each component. This decomposition helps in understanding the variations and trends associated with the COA, EOA, SOA, and WOA over time. By isolating these components, it becomes easier to analyze and interpret the dataset.

**Figure 7:**
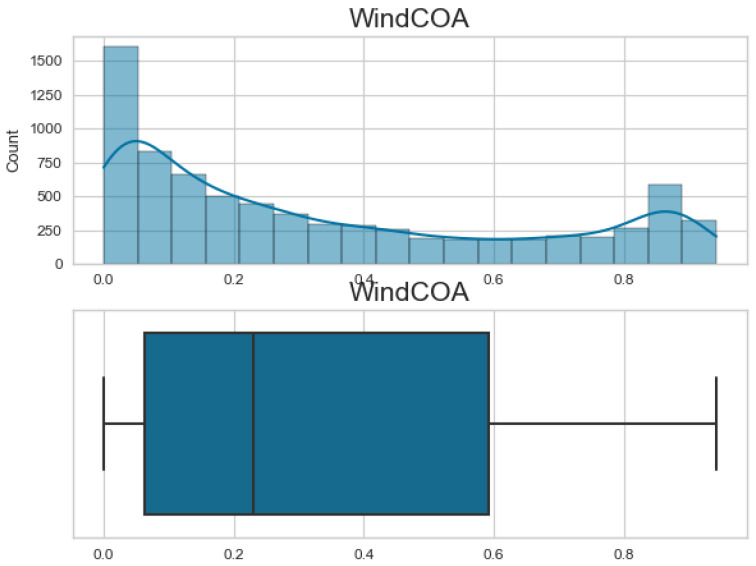
The histogram and box plot for COA of the wind power forecasting dataset.

**Figure 8:**
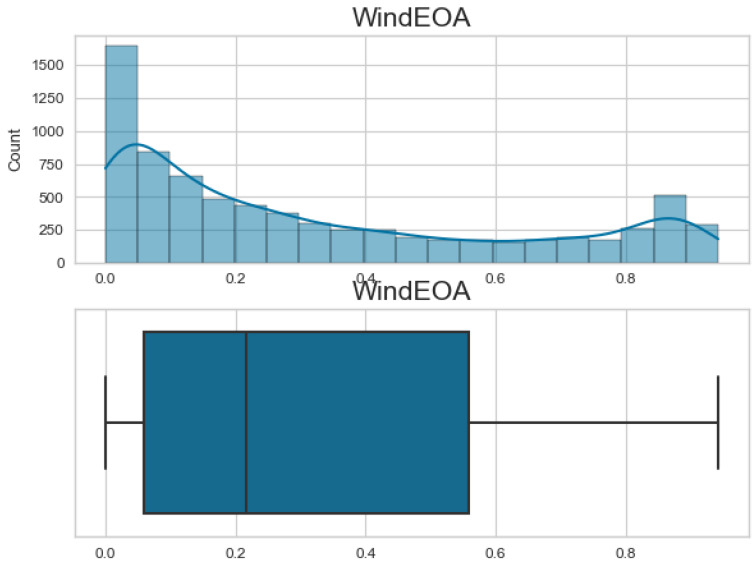
The histogram and box plot for EOA of the wind power forecasting dataset.

**Figure 9:**
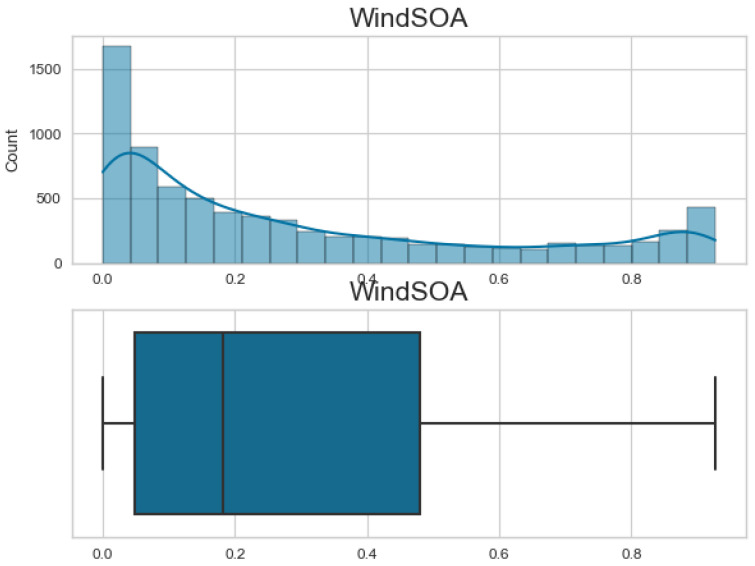
The histogram and box plot for SOA of the wind power forecasting dataset.

**Figure 10:**
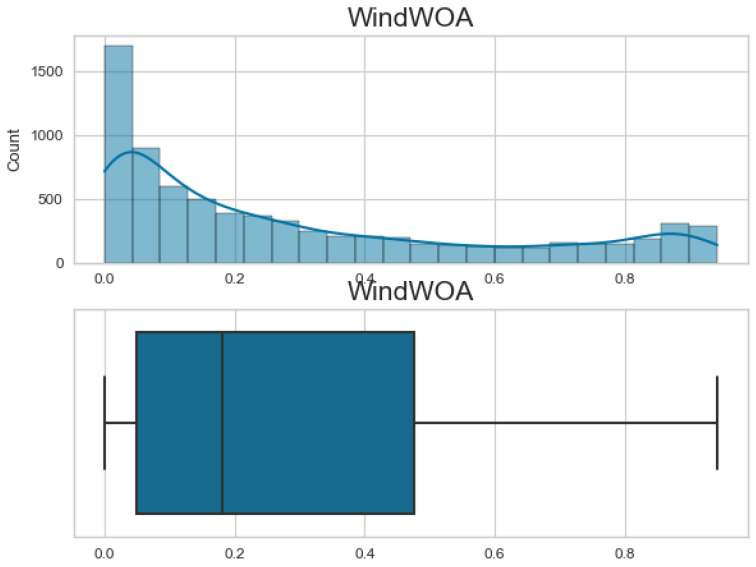
The histogram and box plot for WOA of the wind power forecasting dataset.

Additionally, Figure 7, Figure 8, Figure 9 and Figure 10 display the histogram and box plot for each component. These plots provide a graphical representation of the distribution and statistical properties of the COA, EOA, SOA, and WOA. The histogram gives an overview of the frequency distribution of the values, while the box plot illustrates the median, quartiles, and any outliers present. Together, these figures provide a comprehensive understanding of the wind power forecasting dataset, allowing for a detailed analysis of the COA, EOA, SOA, and WOA components in terms of their seasonal patterns, variations, and statistical characteristics.

### 4.2. Regression Results and Discussions

To assess the effectiveness of the suggested RNN forecasting model based on the DFBER algorithm, various metrics are employed. These metrics encompass relative root mean squared error (RRMSE), Nash Sutcliffe Efficiency (NSE), mean absolute error (MAE), mean bias error (MBE), Pearson’s correlation coefficient (r), coefficient of determination (R2), and determine agreement (WI). The dataset size is denoted by the parameter *N*, while the nth estimated and observed bandwidth values are represented by $\hat{V_{n}}$ and $V_{n}$, respectively. The arithmetic means of the estimated and observed values are denoted as $\bar{\hat{V_{n}}}$ and $\bar{V_{n}}$. The evaluation criteria for predictions can be found in Table 3. The outcomes of the proposed optimizing RNN-DFBER-based model are displayed in Table 4. The results reveal an RMSE value of 0.0028426034752477.

To evaluate the effectiveness of the proposed RNN-DFBER-based model, its regression results are compared with those of other models such as BER, JAYA, FHO, FA, GWO, PSO, GA, and WOA-based models. Table 5 offers a comprehensive statistical description of the RNN-DFBER-based model and presents the RMSE results of all models based on ten independent runs. The description includes information about the minimum, median, maximum, and mean average errors, standard (Std.) Deviation, Std. Error of Mean, the required time in seconds. The Number of values to run the models is ten times.

**Figure 11:**
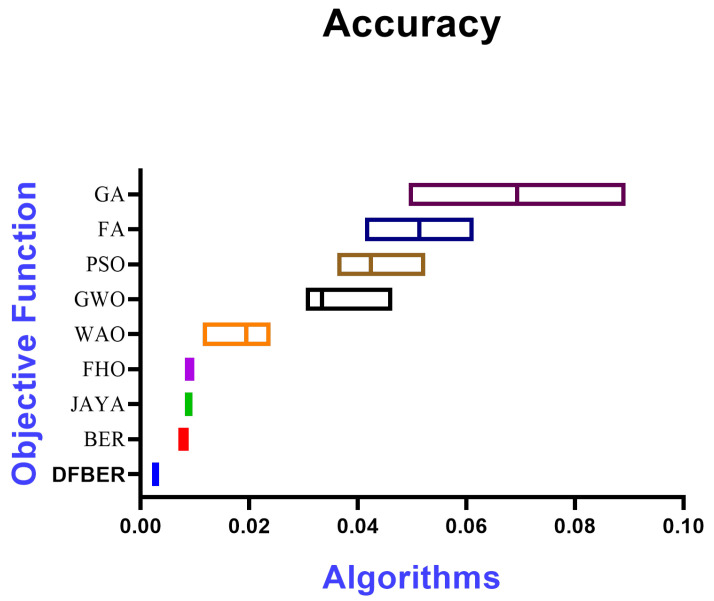
The box plot of the proposed RNN-DFBER-based model and BER, JAYA, FHO, FA, GWO, PSO, GA, and WOA-based models based on the RMSE.

**Figure 12:**
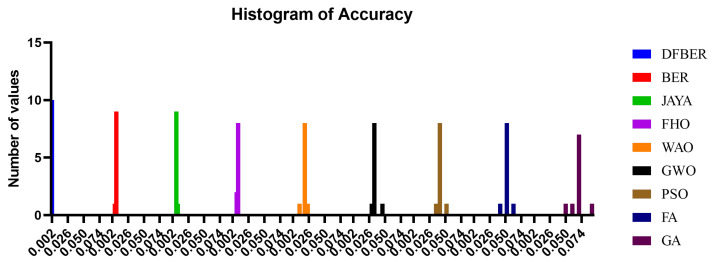
Histogram of RMSE for both the presented RNN-DFBER-based model and the other models.

**Figure 13:**
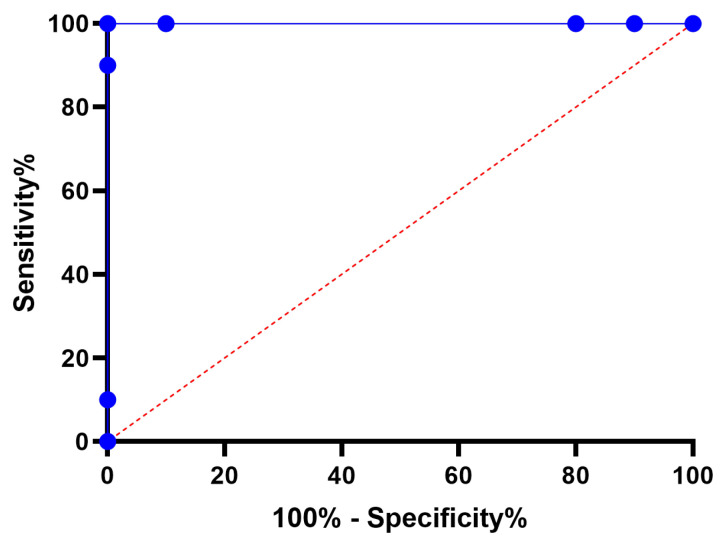
ROC curve of the presented RNN-DFBER algorithm versus the BER algorithm.

**Figure 14:**
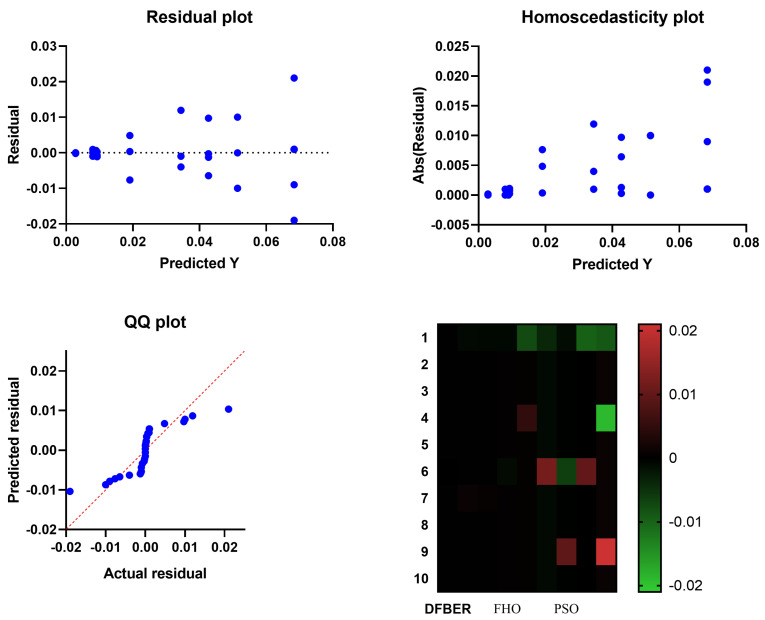
For both the models that were compared and the model that was presented using RNN-DFBER, there were QQ plots, residual plots, and heat maps.

Figure 11 illustrates a box plot generated using the root-mean-squared error (RMSE) for the proposed RNN-DFBER-based model, as well as the BER, JAYA, FHO, FA, GWO, PSO, GA, and WOA-based models. The effectiveness of the optimized RNN-DFBER-based model is assessed using the fitness function described in Equation (10), as depicted in the figure. Additionally, Figure 12 presents a histogram of the RMSE values for both the presented RNN-DFBER-based model and the other models. Furthermore, Figure 13 showcases the ROC curve comparing the presented DFBER algorithm with the BER algorithm. Lastly, Figure 14 showcases QQ plots, residual plots, and a heat map for both the provided RNN-DFBER-based model and the compared models, based on the analyzed data. These figures collectively demonstrate the potential of the given optimized RNN-DFBER-based model to outperform the compared models.

**Figure 15:**
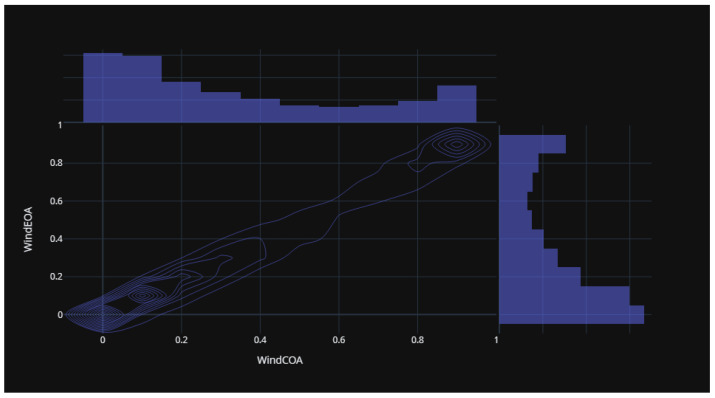
The statistical distribution for COA versus EOA of the wind power forecasting dataset.

**Figure 16:**
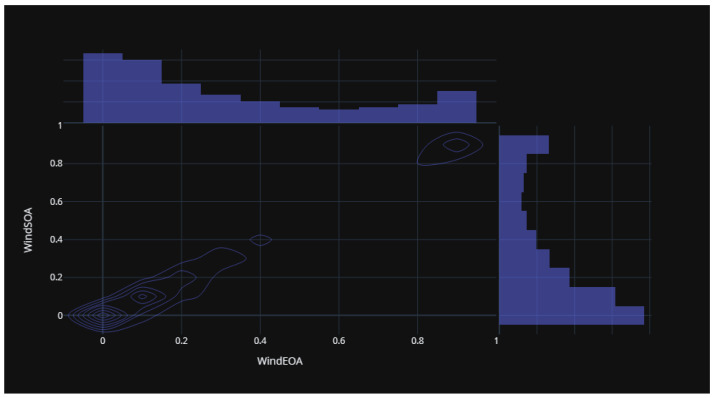
The statistical distribution for EOA versus SOA of the wind power forecasting dataset.

**Figure 17:**
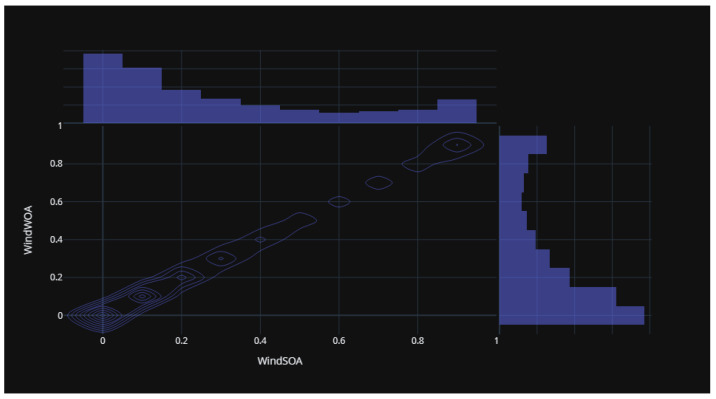
The statistical distribution for SOA versus WOA of the wind power forecasting dataset.

Figure 15 illustrates the statistical distribution of the COA compared to the EOA components of the wind power forecasting dataset. This distribution provides insights into the relationship and variability between these two components. Figure 16 presents the statistical distribution of the EOA in relation to the SOA components of the wind power forecasting dataset. This distribution offers a visualization of the statistical properties and potential correlations between these two components. Lastly, Figure 17 showcases the statistical distribution of the SOA in comparison to the WOA components of the wind power forecasting dataset. This distribution enables an analysis of the statistical characteristics and potential dependencies between these two components. By examining these statistical distributions, one can gain a deeper understanding of the relationships, variations, and patterns present in the wind power forecasting dataset between COA and EOA, EOA and SOA, and SOA and WOA.

The results of the ANOVA test conducted on the proposed RNN-DFBER-based model and the compared models are presented in Table 6. This statistical analysis provides insights into the significance of differences between the models. Additionally, Table 7 presents a comparison between the proposed optimized RNN-DFBER-based model and the compared models using the Wilcoxon Signed-Rank test. By conducting ten independent iterations of each algorithm, the statistical analysis ensures accurate comparisons and enhances the reliability of the study’s findings.

## 5. Discussion

The presented results demonstrate the effectiveness of the proposed optimized RNN-DFBER-based model. It outperforms the compared models based on various evaluation metrics, visual representations, and statistical tests. These findings highlight the potential and superiority of the presented RNN-DFBER-based model in accurately predicting and modeling the data. The model exhibits lower RMSE values, indicating better overall performance in capturing the observed values compared to other models. Furthermore, the comprehensive evaluation using multiple metrics such as RRMSE, NSE, MAE, MBE, r, R2, and WI provides a holistic assessment of the proposed RNN-DFBER-based model. The model’s ability to closely match the observed values, as reflected in the high correlation coefficient (r) and coefficient of determination (R2), demonstrates its effectiveness in capturing the underlying patterns in the data.

The visual comparisons, including the box plot, histogram, ROC curve, QQ plots, residual plots, and heat map, further support the superiority of the proposed RNN-DFBER-based model. These visualizations provide insights into the distribution of errors, discrimination ability, conformity to assumptions, and the relationship between variables, ultimately highlighting the model’s strengths and advantages over the other models. The statistical analyses conducted, including the ANOVA test and the Wilcoxon Signed-Rank test, reinforce the significance and reliability of the findings. By conducting multiple iterations of each algorithm, the comparisons are robust, ensuring that the observed differences in performance are statistically significant.

The results and analyses collectively demonstrate that the proposed optimized RNN-DFBER-based model outperforms the compared models in terms of accuracy, predictive capability, and reliability. These findings contribute to the validation and efficacy of the suggested algorithm for the given dataset and underline its potential for practical applications in similar domains.

## 6. Conclusions and Future Work

In conclusion, the proposed optimized RNN-DFBER-based model has been shown to be highly effective in predicting and modeling the given dataset. The model outperformed other compared models based on various evaluation metrics, including RRMSE, NSE, MAE, MBE, r, R2, and WI. The low RMSE value indicates a close match between the model’s predictions and the observed values. Visual comparisons, such as box plots, histograms, ROC curves, QQ plots, residual plots, and heat maps, further supported the superiority of the RNN-DFBER-based model. These visualizations demonstrated its ability to accurately capture the underlying patterns and relationships in the data. Statistical analyses, including the ANOVA test and the Wilcoxon Signed-Rank test, provided additional evidence of the model’s significance and reliability. By conducting multiple iterations of each algorithm, the statistical comparisons ensured the robustness of the results. The findings indicate that the proposed RNN-DFBER-based model offers a powerful and effective approach for the given dataset. Its strong performance, supported by comprehensive evaluations and statistical analyses, suggests its potential for practical applications in similar domains. Future directions for research in this area could include conducting further research and validation using diverse datasets and scenarios to evaluate the generalizability and robustness of the RNN-DFBER-based model. This would help determine the model’s effectiveness in different contexts and ensure its reliability across various conditions.

## Figures and Tables

**Table 1:** The DFBER algorithm’s configuration settings.

Parameter (s)	Value (s)
Agents	10
Iterations	80
Runs	10
Exploration percentage	70%
*K* (decreases from 2 to 0)	1
Mutation probability	0.5
Random variables	[0, 1]

**Table 2:** Compared algorithms various configuration parameters.

Algorithm	Parameter (s)	Value (s)
BER	Mutation probability	0.5
	Exploration percentage	70%
	*k* (decreases from 2 to 0)	1
	Agents	10
	Iterations	80
JAYA	Population size	10
	Iterations	80
FHO	Population size	10
	Iterations	80
FA	Gamma	1
	Beta	2
	Alpha	0.2
	Agents	10
	Iterations	80
GWO	*a*	2 to 0
	Wolves	10
	Iterations	80
PSO	Acceleration constants	[2, 2]
	Inertia $W_{min}$, $W_{max}$	[0.6, 0.9]
	Particles	10
	Iterations	80
GA	Cross over	0.9
	Mutation ratio	0.1
	Selection mechanism	Roulette wheel
	Agents	10
	Iterations	80
WOA	*r*	[0, 1]
	*a*	2 to 0
	Whales	10
	Iterations	80

**Table 3:** Evaluation criteria for predictions.

Metric	Formula
RMSE	$\sqrt{\frac{1}{N}\sum_{n=1}^{N}(\hat{V_{n}}-V_{n}{)}^{2}}$
RRMSE	$\frac{RMSE}{\sum_{n=1}^{N}\hat{V_{n}}}\times 100$
MAE	$\frac{1}{N}\sum_{n=1}^{N}\left|\hat{V_{n}}-V_{n}\right|$
MBE	$\frac{1}{N}\sum_{n=1}^{N}\left(\hat{V_{n}}-V_{n}\right)$
NSE	$1-\frac{\sum_{n=1}^{N}(V_{n}-\hat{V_{n}}{)}^{2}}{\sum_{n=1}^{N}(V_{n}-\bar{\hat{V_{n}}}{)}^{2}}$
WI	$1-\frac{\sum_{n=1}^{N}\left|\hat{V_{n}}-V_{n}\right|}{\sum_{n=1}^{N}|V_{n}-\bar{V_{n}}\left|+\right|\hat{V_{n}}-\bar{\hat{V_{n}}}|}$
R2	$1-\frac{\sum_{n=1}^{N}(V_{n}-\hat{V_{n}}{)}^{2}}{\sum_{n=1}^{N}(\sum_{n=1}^{N}V_{n})-V_{n}{)}^{2}}$
r	$\frac{\sum_{n=1}^{N}\left(\hat{V_{n}}-\bar{\hat{V_{n}}}\right)\left(V_{n}-\bar{V_{n}}\right)}{\sqrt{\left(\sum_{n=1}^{N}(\hat{V_{n}}-\bar{\hat{V_{n}}}{)}^{2}\right)\left(\sum_{n=1}^{N}{\left(V_{n}-\bar{V_{n}}\right)}^{2}\right)}}$

**Table 4:** Proposed RNN-DFBER-based model results.

	RMSE	MAE	MBE	r	R2	RRMSE	NSE	WI
RNN-DFBER	0.002843	0.000230	0.000596	0.999325	0.999106	0.301785	0.999106	0.998771

**Table 5:** Statistical description of the proposed RNN-DFBER-based model and other models’ results from RMSE.

	RNN-DFBER	RNN-BER	RNN-JAYA	RNN-FHO	RNN-WOA	RNN-GWO	RNN-PSO	RNN-FA
Number of values	10	10	10	10	10	10	10	10
Minimum	0.002643	0.006966	0.008195	0.00815	0.01146	0.03041	0.03624	0.04138
Maximum	0.002943	0.008897	0.009595	0.009498	0.02395	0.04634	0.0524	0.06138
Range	0.0003	0.001931	0.0014	0.001348	0.01249	0.01593	0.01616	0.02
Mean	0.002823	0.007959	0.00894	0.009263	0.0191	0.0344	0.04268	0.05138
Std. Deviation	7.89 × 10^−5^	0.000455	0.000331	0.000502	0.003035	0.004299	0.003918	0.004714
Std. Error of Mean	2.49 × 10^−5^	0.000144	0.000105	0.000159	0.00096	0.001359	0.001239	0.001491
Time(S)	17.85	23.56	29.15	31.24	33.68	35.45	37.87	38.17

**Table 6:** The outcomes of the ANOVA test for the comparison models and the suggested RNN-DFBER.

	SS	DF	MS	F (DFn, DFd)	*p* Value
Treatment (between columns)	0.04257	8	0.005321	F (8, 81) = 290.7	*p* < 0.0001
Residual (within columns)	0.001483	81	0.0000183	-	-
Total	0.04405	89	-	-	-

**Table 7:** Comparison between the models that were compared using the Wilcoxon Signed-Rank test and the proposed RNN-DFBER.

	DFBER	BER	JAYA	FHO	WOA	GWO	PSO	FA	GA
Theoretical median	0	0	0	0	0	0	0	0	0
Actual median	0.002843	0.007966	0.008951	0.009498	0.01946	0.03341	0.0424	0.05138	0.06938
Number of values	10	10	10	10	10	10	10	10	10
Wilcoxon Signed Rank Test									
Sum of signed ranks (W)	55	55	55	55	55	55	55	55	55
Sum of positive ranks	55	55	55	55	55	55	55	55	55
Sum of negative ranks	0	0	0	0	0	0	0	0	0
*p* value (two tailed)	0.002	0.002	0.002	0.002	0.002	0.002	0.002	0.002	0.002
Exact or estimate?	Exact	Exact	Exact	Exact	Exact	Exact	Exact	Exact	Exact
Significant (alpha = 0.05)?	Yes	Yes	Yes	Yes	Yes	Yes	Yes	Yes	Yes
How big is the discrepancy?									
Discrepancy	0.002843	0.007966	0.008951	0.009498	0.01946	0.03341	0.0424	0.05138	0.06938

## Data Availability

King Abdullah Petroleum Studies and Research Center. Available online: https://datasource.kapsarc.org/explore/dataset/ wind-solar-energy-data/information/ (accessed on 1 June 2023).
